# 

**Published:** 1900-05-12

**Authors:** 


### NOTICE TO CORRESPONDENTS.

All MS., Letters, Books for Review, and other matters intended for the Editor should be addressed THE EDITOR, "THE HOSPITAL," THE HOSPITAL BUILDING, 28 & 29, SOUTHAMPTON STREET, STRAND, W.C.

The Editor cannot undertake to return rejected MS., even when accompanied by stamped directed envelope.

**Notice to Advertisers and Subscribers.**

All Advertisements, Orders for Copies of THE HOSPITAL, and Business Communications should be addressed to **THE MANAGER (Not to the Editor),** THE HOSPITAL, The Hospital Building, 28 & 29, Southampton Street, Strand, London, W.C.

The Cost of Subscription, post free, is as follows:—Per week, 2½d.; per quarter, 2s. 8d.; per half-year, 5s. 3d.; per annum, 10s. 6d. Foreign:—Per annum, 15s. 2d., payable, in all cases, in advance.

**NOTICE.—Vol. XXVII. of "The Hospital" (October, 1899, to April, 1900), in handsome cloth binding, is now on sale at the Office of this Journal, price 7s. 6d. Cases for binding the Numbers contained in this Volume 1s. 6d. Index to Vol. XXVII., 2½d., post free.**

**The Monthly Part for May is now ready. Price 6d., by post 8d.**

### The Student of Medicine.

### APPOINTMENTS.

J. Armstrong, L.R.C.P., L.R.C.S.Irel., appointed Certifying Factory Surgeon for the Ballymahon Dispensary District, co. Longford. E. H. Binney, M.B. and Ch.M.Sydney, appointed Honorary Surgeon at the Hospital for Sick Children, Glebe Point, Sydney, New South Wales. A. H. Buchan, M.A., M.B., C.M., M.R.C.P.E., appointed an Assistant Physician to the Leith Hospital. J. Eason, M.B., C.M., M.R.C.P.E., appointed Assistant Physician and Registrar to the Leith Hospital. J. Elliott, L.R.C.P., L.R.C.S.Edin., appointed Medical Officer for the Islandshire District of the Berwick-on Tweed Union. P. H. Hadfield, M.A., L.R.C.S.&P.E., L.F.P.S.G., appointed Senior House Surgeon to the Stanley Hospital, Liverpool. G. Hamilton, M.B., Ch.B.Edin., appointed Senior House Surgeon to the Children's Infirmary, Liverpool. C. O. Hawthorne, M.D., M.R.C.P., appointed Assistant Physician to the Royal Hospital for Children and Women, Waterloo Bridge Road, London. G. Lys, M.R.C.S.Eng., L.R.C.P.Lond., appointed Medical Officer for the Bere Regis District of the Wareham and Purbeck Union. W. J. Mackinnon, M.B., C.M.Glasg., appointed Certifying Factory Surgeon for the Biggar District, co. Lanark. F. Nolans, L.R.C.P., L.R.C.S.Irel., appointed Certifying Factory Surgeon for the Gorey Dispensary District, co. Wexford. H. B. Smith, M.B., C.M.Glasg., appointed Junior Assistant Medical Officer to the County Asylum, Bicton Heath, near Shrewsbury. E. M. Sympson, M.A., M.D., B.C.Cantab., M.R.C.S., appointed Honorary Physician to the Lincoln and Lincolnshire Home for Girls. L. L. Thain, M.R.C.S.Eng., appointed Medical Officer for the Kentchurch District and the Workhouse of the Dore Union. F. J. Walker, M.D., appointed Coroner for the Spilsby Division of Lincolnshire. R. E. H. Woodforde, M.R.C.S., L.R.C.P., appointed Medical Officer for the Third District of the Royston Union.

28 & 29, SOUTHAMPTON STREET, STRAND, LONDON, W.C.

Hovis Bread

(TRADE MARK.)

Is a tasty and digestive bread, most suitable for convalescents and dyspeptics.

Most Family Bakers make and deliver it daily. Address of Agents will be sent by The Hovis-Bread Flour Co. Ld., Macclesfield, on application.

Accouchement Requisites.

Ambulance Cases.

### NOW READY,

A REPRINT OF

### A NURSING HANDBOOK

TOGETHER WITH S. MAW, SON, AND THOMPSON'S

### COMPLETE CATALOGUE OF NURSING REQUISITES.

The above work is now republished at a cost of 1s. per copy. Messrs. S. M., S., and T. have been compelled to make this charge on account of the **extreme** popularity of the work and the great expense incurred in its production.

***Post Free on receipt of 1s.***

### S. MAW, SON, and THOMPSON,

7 to 12, ALDERSGATE STREET, LONDON, E.C.

### BOOKS ON NURSING.

**A COMPLETE SYSTEM OF NURSING.** Written by various contributors, consisting entirely of Medical Men and Nurses. Edited by HONNOR MORTEN, Author of "The Nurse's Dictionary," &c. 8vo., cl., 7s. 6d. *net.*

"We are bound to say that the greater part of this handy volume contains much that is useful and interesting. . . . Lastly, the list of recipes for invalid cookery is both original and serviceable."—*The Nurses' Journal.*

"As a book of reference, a nurse may find in its pages exactly the information she may want to resolve a doubt or difficulty."—*Glasgow Herald.*

"The chapter on 'The Nursing of Babies' is admirable, and should be read by all mothers as well as nurses."—*The Hospital.*

**WOUNDED IN WAR.** Surgeon's Handbook of Treatment. By F. ESMARCH, translated by H. H. CHILTON. With coloured plates and 536 engravings. 8vo., £1 8s.

**MANUAL OF FIRST AID.** Being a Text-book for Ambulance Classes and a Work of Reference for Domestic and General Use. By Dr. J. A. AUSTIN, Lecturer to St. John's Ambulance Association, and Author of "Ambulance Sermons." Illustrated with diagrams. Dedicated by permission to H.R.H. Princess Christian of Schleswig-Holstein. Crown 8vo., 3s. 6d. [*Ready,*

"Dr. Austin's little book is well and interestingly written. . . . The plan of the book follows the syllabus of the St. John's Ambulance Association, and is copiously illustrated with diagrammatic figures, which are plain and simple and accurate. Altogether the book is a good specimen of its class."—*Lancet.*

**HOW TO TREAT ACCIDENTS AND ILLNESSES.** A Handbook for the Home. By HONNOR MORTEN, Author of "Sketches of Hospital Life," &c. Second Edition. With illustrations. Post 8vo., boards, 2s.; cloth, 2s. 6d.

**LYING-IN.** A Book for Every Mother. By MARIAN HUMFREY, of the British Lying-In Hospital, London, and Author of "A Manual of Obstetric Nursing." Small crown 8vo., paper covers, 1s.

DR. ALFRED HILL (Medical Officer of Health, Birmingham) says:—"I have gone through the book, and am convinced that if lying-in women will follow its directions, the discomforts, dangers, and diseases which too often attend the natural process will be either avoided or mitigated."

**1. A MANUAL OF OBSTETRIC NURSING.** By MARIAN HUMFREY. Part I.—MATERNAL. Part II.—INFANTILE. Crown 8vo., cloth, 3s. 6d. Dedicated by permission to H.R.H. the late Princess Mary of Cambridge, Duchess of Teck.

*The Hospital* says: "This volume is certainly a very valuable addition to the literature of obstetric nursing. Not only is every little detail mentioned, but its special excellence consists in the very lucid manner in which all is explained. . . . The arrangement of the chapters, and the advice given are beyond praise."

**2. A MANUAL OF OBSTETRIC NURSING.** By MARIAN HUMFREY. Vol. II. Part I.—MATERNAL. Part II.—INFANTILE. Crown 8vo., cloth, 3s. 6d. This volume treats of duties, special to the gravest emergencies, of Obstetric Nursing, and thus completes the curriculum of instruction.

LONDON: SAMPSON LOW, MARSTON & COMPANY, LIMITED., St. Dunstan's House, Fetter Lane, E.C.

